# Preclinical efficacy and toxicity studies of a highly specific chimeric anti‐CD47 antibody

**DOI:** 10.1002/2211-5463.13084

**Published:** 2021-02-22

**Authors:** Zhiqiang Xu, Jing Gao, Jingyun Yao, Teddy Yang, Dongxu Wang, Chaohui Dai, Yu Ding

**Affiliations:** ^1^ School of Life Sciences Fudan University Shanghai China; ^2^ Biologics Discovery Shanghai ChemPartner Co., Ltd Shanghai China; ^3^ Biologics Discovery Shanghai Hyamab Biotechnology Co., Ltd Shanghai China

**Keywords:** 4D10, antibody, CD47, haemagglutination, phagocytosis, SIRPα

## Abstract

Cluster of differentiation 47 (CD47) is a widely expressed self‐protection transmembrane protein that functions as a critical negative regulator to induce macrophage‐mediated phagocytosis. Overexpression of CD47 enables cancer cells to escape immune surveillance and destruction by phagocytes both in solid tumours and leukaemia. The usefulness of anti‐CD47 antibody has been demonstrated in multiple immunotherapies associated with macrophages. However, antigen sinks and toxicity induced by inadvertent binding to normal cells restrict its clinical applications. Here, a novel anti‐human CD47 antibody, 4D10, was generated, and its variable regions were grafted onto a human IgG4 scaffold. Compared with the anti‐CD47 antibody Hu5F9, the resulting chimeric antibody (c4D10) has consistently demonstrated good tolerance in *in vitro* and *in vivo* toxicity studies. Additionally, c4D10 showed effective therapeutic potential through inducing the eradication of human cancer cells. Thus, c4D10 is a promising candidate therapeutic antibody with higher efficacy and reduced side effects compared to earlier antibodies, and its use may reduce the dose‐limiting toxicity of CD47 antagonists for immunotherapy.

## Graphical abstract

Cluster of differentiation 47 (CD47) is a critical negative regulator with respect to inducing macrophage‐mediated phagocytosis. Overexpression of CD47 enables cancer cells to escape immune surveillance and destruction by phagocytes. A novel anti‐CD47 antibody, c4D10, showed effective therapeutic potential through inducing the eradication of human cancer cells (A) and demonstrated good tolerance in *in vitro* and *in vivo* toxicity studies (B).

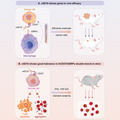

**Abbreviations**
AML: acute myeloid leukaemia
CD47: cluster of differentiation 47
CFSE: carboxyfluorescein succinimidyl ester
FACS: fluoresence‐activated cell sorting
PBMC: peripheral blood mononuclear cells
RBC: red blood cell
SIRPα: signal regulatory protein‐alpha

Cluster of differentiation 47 (CD47), also known as integrin‐associated protein, is a widely expressed cell membrane receptor belonging to the immunoglobulin superfamily. It is regarded as a self‐protection transmembrane protein in normal cells that resists the elimination of macrophage‐mediated phagocytosis. Upon binding to signal regulatory protein‐alpha (SIRPα), CD47 triggers a phosphorylation cascade of the immunoreceptor tyrosine‐based inhibition motif on the cytoplasmic tail of SIRPα, which acts as a ‘don't eat me’ signal [1]. Nevertheless, in multiple haematologic and solid malignancies, the expression of CD47 is abnormally upregulated compared to that in corresponding normal cells [2, 3]. Moreover, numerous studies demonstrated that high CD47 expression was correlated with poor disease survival, indicating that CD47 could act as an adverse prognostic factor in numerous cancers, including myeloid leukaemia, prostate carcinoma, lung carcinoma, breast carcinoma and hepatocellular cancer [4, 5, 6, 7]. Indeed, pathological studies have consistently indicated that the expression of CD47 is directly regulated by the MYC proto‐oncogene, suggesting that CD47 functions as a pro‐tumorigenic factor [8].

More recently, after the molecular mechanism of the role of CD47 in the process of tumour cell escape from immune recognition was elucidated, targeting CD47 has become a novel approach for treatment and has changed the method of cancer immunotherapy. Through directly induced tumour cell death and activated phagocytosis of macrophages [9], the blocking of CD47 effectively facilitated the eradication of tumour cells and the subsequent cross‐priming of tumour‐specific cytotoxic T cells to activate the adaptive immune response [10]. Furthermore, other immune cells, such as natural killer cells, granulocytes and dendritic cells, may also respond to CD47/SIRPα blocking therapies [11, 12, 13, 14]. By recruiting additional immune cells to tumours and synergizing the response of innate and adaptive immunity, CD47 blockade has displayed tremendous pharmacological advantages for preventing tumour recurrence and treating advanced‐stage malignancies and complications [15].

Currently, 10 CD47 antibodies and four SIRPα fusion proteins are being evaluated for clinical efficacy in various type of cancer [16]. As a result of the ubiquitous expression and unique properties of CD47, more attention is now principally being focused on haematotoxicity, including anaemia and thrombocytopenia, caused by the administration of anti‐CD47 blocking antibodies in clinical oncology studies [17, 18, 19]. To minimize adverse events and release antigen‐independent therapeutic effects, most CD47 antagonists have been grafted to the IgG4 subclass with low affinities for FcγRIIa and FcγRIIIa, which mediate antibody‐dependent cellular phagocytosis of phagocytes and antibody‐dependent cellular cytotoxicity of natural killer cells, respectively [20]. However, abrogating the effector functions of the competent Fc region may result in an unpredictable decline in the therapeutic response. Indeed, Celgene has terminated a phase 1 study of CC‐90002, as an agent of monotherapy, as a result of an insufficiently encouraging profile for further dose escalation/expansion in relapsed/refractory acute myeloid leukaemia (AML) and high‐risk myelodysplastic syndromes (www.clinicaltrials.gov identifier: NCT02641002). Meanwhile, Hu5F9‐G4 combined with rituximab, an anti‐CD20 conventional cancer cell‐opsonizing antibody, achieved an exciting goal in patients with relapsed/refractory diffuse large B‐cell lymphoma and indolent non‐Hodgkin lymphoma [21]. Therefore, there is still an ongoing and urgent need to exploit a safe and highly specific anti‐CD47 antibody as a single agent or optional combination treatment.

In the present study, we developed an IgG4 subclass chimeric anti‐CD47 antibody, termed c4D10, based on hybridoma technology. It displayed biological activity comparable to reference molecules (Hu5F9‐G4 and CC‐90002) during *in vitro* studies. Meanwhile, c4D10 elicited significant, potent macrophage‐mediated phagocytosis of tumour cells in non‐obese diabetic/severe combined immunodeficient (NOD/SCID) mice. Moreover, it exhibited a satisfactory safety profile, such that it did not cause T‐cell death or haemagglutination *in vitro* and demonstrating limited haematologic toxicity *in vivo*. In haematological analysis, except for a mild red blood cell (RBC) and haemoglobin drop, no notable effect on serum biochemistry and other haematocytes was observed in the hCD47/hSIRPα double knock‐in model administered with this anti‐CD47 blocking antibody. Overall, c4D10 comprises a promising candidate in the exploitation of novel anti‐CD47 therapeutic agents.

## Materials and methods

### Cells and proteins

The human CD47 or human SIRPα gene encoding the extracellular domain was fused to a Fc tag and cloned into pCPC vector (plasmid was prepared from pCEP4; Invitrogen, Carlsbad, CA, USA), followed by transiently transfecting HEK293 cells (Invitrogen) for fusion protein production. The anti‐CD47 antibodies CC‐90002 and Hu5F9‐G4 were generated internally based on publicly available sequences. The recombinant proteins and antibodies were purified from supernatant by protein A affinity chromatography and Superdex 200 (GE Healthcare, Chicago, IL, USA) size exclusive chromatography. The molecular weight and purity of the target proteins were verified by SDS/PAGE and mass spectrometry.

The nucleotide sequences encoding the full‐length amino acid sequence of human or cynomolgus CD47 were cloned into the pLVX‐IRES vector (Clontech, Mountain View, CA, USA). Stable cell lines (CHOK1/hCD47 and CHOK1/cynoCD47) were constructed by transiently transfecting CHOK1 cells with the respective plasmids followed by subsequent selection with Ham's F‐12K (Kaighn's) medium (Gibco, Waltham, MA, USA) supplemented with 10% FBS (Biological Industries, Beit Haemek, Israel), 6 μg·mL^−1^ puromycin (Invitrogen) and 1% Pen‐strep solution (Biological Industries) at 37 °C in a 5% CO_2_ incubator.

Human peripheral blood mononuclear cells (PBMCs) were separated from whole blood by centrifugation at 400 ***g*** for 30 min on a Ficoll‐Paque (GE Healthcare) density gradient and human RBCs were isolated from whole blood by centrifugation at 200 ***g*** for 10 min at room temperature with the brake turned off. All studies on human materials were approved by the Institutional Ethics Committee (IEC) of ChemPartner (Shanghai, China) (IEC protocol NO: IEC001‐R2015) and written informed consent was obtained from all donors.

### Generation of murine antibodies

For generation of murine antibodies, 6–8‐week‐old SJL mice (SLAC Laboratory, Menlo Park, CA, USA) were immunized with the purified hCD47/Fc fusion protein with an interval of 2 weeks between the initial immunization and the first booster immunization, and a 3‐week interval between subsequent booster immunizations for a total of 5 weeks. Blood was collected 1 week after each boost, and the antibody titre and specificity of the immunogen in the serum were measured by flow cytometry. When sufficient antibody titre was reached in serum, immunized mice were scarified and the spleen cells were fused with SP2/0 cells. Hybridomas were selected and supernatants from the resulting clones were screened by an ELISA and fluorescence‐activated cell sorting (FACS).

### Variable region cloning and sequencing

After the supernatant obtained from the subcloning culture was tested, 5 × 10^7^ hybridoma cells were collected and prepared for initial RNA isolation using an RNeasy Plus Mini Kit (Qiagen, Hilden, Germany). Total cDNA synthesis with a specific sequence at the 5′ end was performed with PrimeScript RT Master Mix (Takara, Kyoto, Japan) utilizing the extracted RNA as a template. Next, cDNA containing the whole variable region from hybridoma cell lines was amplified and cloned into a TA vector. The DNA sequences of the variable regions of mAbs were analysed by DNA sequencing.

### Preparation of human‐mouse IgG4 chimeric anti‐CD47 monoclonal antibodies

Based on the DNA sequences of variable regions, chimeric antibodies were cloned into a pCPC vector by connecting the variable regions of the mouse hybridoma mAbs to the constant region of human IgG4PE (S228P/L235E) kappa containing a signal peptide by overlapping PCR, with confirmation by DNA sequence analysis. Finally, the heavy and light chain IgG expressing vectors were transiently co‐transfected into FreeStyle 293‐F cells (Invitrogen) for further production of chimeric antibodies. The name of each chimeric antibody is defined by the corresponding antibody clone number with the initial character ‘c’.

### Cell surface antigen‐binding assay

For cell surface antigen‐binding assay, CHOK1/hCD47 or CHOK1/cynoCD47 cells were plated in 96‐well plates at a density of 3 × 10^5^ cells per well; RBCs were seeded into 96‐well plates at a density of 2 × 10^6^ cells per well. Various concentrations (from 200 nm) of anti‐CD47 antibodies or isotype control were added and incubated with the cells at 4 °C for 1 h. After removing the antibodies, cells were stained with a 1 : 1000 dilution of Alexa Fluor 488 goat anti‐human IgG (Invitrogen), washed and then fixed in 0.4% paraformaldehyde (Boster Biological Technology, Pleasanton, CA, USA). Flow cytometry was performed on a FACS Canto II flow cytometer (BD Biosciences, Franklin Lakes, NJ, USA). Data were analysed using flowjo (Treestar Inc., Ashland, OR, USA).

### Antibody affinity measurement

The binding kinetics of anti‐CD47 candidate mAbs to CD47 was evaluated by a label‐free bio‐layer interferometry assay on an Octet Red 384 (ForteBio, Fremont, CA, USA). All experiments were performed at 25 °C in PBS with 0.005% Tween‐20. Candidate mAbs were loaded onto anti‐hIgG Fc capture sensors at a concentration of 5 μg·mL^−1^ in Stage 1 and CD47‐His (Sino Biology, Beijing, China) was loaded onto the biosensor for 600 s to obtain saturation in Stage 2. All sensors were regenerated using 10 mm glycine‐HCl buffer (pH 1.7) (GE Healthcare). The data collected were processed and analysed using octet data analysis (ForteBio).

### HCD47/hSIRPα interaction blocking assay

The blocking activity of CD47 lead candidate antibody was evaluated by a competitive ELISA. Briefly, 96‐well plates were coated with 1 μg·mL^−1^ SIRPα‐hFc in PBS at 100 μL per well at 4 °C overnight, followed by blocking with 1% BSA (Amresco, Solon, OH, USA). The indicated concentrations of candidate mAbs (up to 200 nm) were applied to the ELISA plate containing 0.005 μg·mL^−1^ biotin‐conjugated human CD47 and immobilized human SIRPα, and incubated for 1 h at 37 °C. Bound protein (biotinylated human CD47) was detected with a horseradish peroxodase‐conjugated secondary antibody specific to streptavidin (Sigma, St Louis, MO, USA). The addition of 3,3′,5,5′‐tetramethylbenzidine substrate produced optical densities proportional to bound antibody and was measured using a SpectraMax M5 Multi‐mode Plate Reader (Molecular Devices, San Jose, CA, USA). Four parameter fit curves were generated with prism, version 6 (GraphPad Software Inc., San Diego, CA, USA).

### Phagocytosis assay

After isolation over a Ficoll‐Paque Plus density gradient, human PBMCs were plated in a tissue culture dish (100 × 20 mm) (Corning Inc., Lowell, MA, USA) with basal culture medium RPMI‐1640 (Gibco), at a concentration of 2 × 10^6^ cells·mL^−1^ for 2 h in a humidified incubator with a 5% CO_2_ atmosphere at 37 °C. Adherent monocytes were stimulated with macrophage colony‐stimulating factor (PeproTech, Rocky Hill, NJ, USA) for 7–10 days to obtain macrophages. Macrophages were harvested and cocultured with carboxyfluorescein succinimidyl ester (CFSE)‐labelled (Sigma) Jurkat cells (ATCC, Manassas, VA, USA) in the presence of serial dilutions of candidate mAbs (from 0.004 to 66.67 nm) in the ratio 1 : 4 for 4 h. Macrophages were then stained with anti‐CD14 (eBioscience, San Diego, CA, USA) and FACS was performed. The CD14^+^CFSE^+^ population represented phagocytic cells.

### AML xenograft model in NOD/SCID mice

Raji cells (CCL‐86; purchased from ATCC) were maintained *in vitro* as a suspension culture at a density of 4 × 10^5^ cells ml^–1^ in RPMI‐1640 medium enriched with 10% FBS and 1% Pen‐strep solution at 37 °C in the 5% CO_2_ incubator. The tumour cells were routinely subcultured twice weekly. When the tumour size reached 79.97 mm^3^ for the tumour efficacy study (day 14 post‐inoculation), 24 tumour‐bearing mice were block randomized into three groups with eight mice in each group. All mice were treated with 10 mg·kg^−1^ anti‐CD47 antibodies or control intravenously on days 1, 3, 5, 8, 10, 12, 15, 17 and 19. Animal weight was recorded and the tumour size was measured in two dimensions using a calliper twice weekly. The tumour volume (mm^3^) was expressed using: *V* = 0.5 × *a* × *b* ^2^, where *a* and *b* are the long and short diameters of the tumour, respectively. On day 25 after the mice received tumour cells, all mice were killed and tumours were harvested and weighed.

The present study was carried out in accordance with the protocol approved by the Institutional Animal Care and Use Committee of Shanghai Chempartner (IACUC protocol No. B11‐20180620‐0001‐20210620) following the guidance of the Association for Assessment and Accreditation of Laboratory Animal Care. Animals (certificate number: SCXK (Ze) 2019‐0001 1911280060) that were observed to be in a continuously deteriorating condition or with a tumour size exceeding 20% body weight were euthanized.

### Haemagglutination assay

For the haemagglutination assay, human RBCs were mixed with PBS to generate a 5% (V/V) cell suspension and seeded to a round‐bottom 96‐well plate. Serial dilutions of candidate mAbs (from 0.01 to 333.33 nm) were added and incubated with human erythrocytes for 2–6 h at room temperature. Haemagglutination was determined by the presence of non‐settled RBCs, appearing as a haze compared to the punctuated red dots of non‐haemagglutinated RBCs. The haemagglutination score was determined by quantifying the area of the RBC pellet in the presence of the antibody, normalized to that in the absence of the antibody.

### Apoptosis assay

Ninety‐six‐well plates were coated with 1 μg·mL^−1^ anti‐CD3 (ChemPartner) and serial dilutions of candidate mAbs (from 0.67 to 66.67 nm) in PBS for 16 h at 4 °C, followed by washing with PBS three times. Primary T cells were separated from human PBMCs by positive selection (EasySep Human CD3^+^ T Cell Isolation Kit; Stemcell Technologies, Vancouver, BC, USA) in accordance with the manufacturer’s instructions. Then, 3 × 10^5^ CD3^+^ T cells in RPMI‐1640 (Gibco) containing 10% FBS were added per well to the coated 96‐well plates followed by incubation overnight. The next day, cells were harvested and stained with Annexin‐V‐Alexa 488 (Invitrogen) for apoptosis cell counting.

### Toxicity study in hCD47/hSIRPα double knock‐in mice

The *in vivo* toxicity of the anti‐CD47 antibody was evaluated in the hCD47/hSIRPα double knock‐in model. All hCD47/hSIRPα double knock‐in mice, from Beijing Biocytogen Co., Ltd (Beijing, China) with certificate number SYXK (Su) 2016‐0004, were randomly assigned to one of four groups (three mice per group) and injected intraperitoneally with 10 mg·kg^−1^ anti‐CD47 antibody or control on days 0, 2 and 4. Animal weight was measured, serum were prepared for blood chemistry tests on days 6, and whole blood was analysed for complete blood counts on days 1, 3, 6 and 13.

The animal study was approved by the Biocytogen Animal Care Committee in accordance with the regulations of the IACUC. At the time of routine monitoring, any observed adverse clinical signs were described and documented in the study record. Animals with severe clinical abnormal signs, with no improvement, or animals not anticipated to recover before the next scheduled time point or dose administration were euthanized.

## Results

### Generation of chimeric monoclonal antibodies against human CD47

In the present study, a hCD47/Fc fusion protein was utilized to immunize SJL mice and produce monoclonal mouse anti‐human CD47 antibodies. After the binding activities with CHOK1/hCD47 and hCD47/hSIRPα blocking activity were estimated, one of the high specific positive clones was obtained and designated as 4D10. Using standard molecular biology cloning techniques, heavy and light variable regions of 4D10 were routinely cloned and sequenced for further analysis. In the amino acid sequence alignment BLAST results, the sequence of 4D10 displayed 64% homology with that of Hu5F9‐G4 produced by Forty Seven (Menlo Park, CA, USA) and 54% homology with CC‐90002 of Celgene in the VL region, and the homology of the VH region of 4D10 with Hu5F9‐G4 and CC‐90002 was 69% and 57%, respectively (Table 1). As a potential therapeutic antibody, no post‐translational modification hotspot, such as Asn‐Gly (NG) motif, Asp‐Gly (DG) motif, Asn‐X‐Ser/Thr (NXS/T) motif and free cystine, was included in the variable regions [22, 23, 24]. All of the results above support the hypothesis that 4D10 is a novel and structurally stable antibody molecule. Finally, variable regions of 4D10 were genetically fused to a human IgG4 backbone that recruits fewer Fc‐dependent effector mechanisms compared to different human IgG subclasses. The human IgG4 CH1 domain was also modified to incorporate the Ser228Pro substitution to minimize the rate of half‐molecule exchange (‘Fab‐arm exchange’) [25, 26, 27].

**Table 1:** BLAST results of c4D10 with reference antibody sequences.

Antibody region	Hu5F9‐G4	CC‐90002
VL	64%	54%
VH	69%	57%

### Characterization of antigen binding activity of c4D10 by flow cytometry and bio‐layer interferometry

**Fig. 1:**
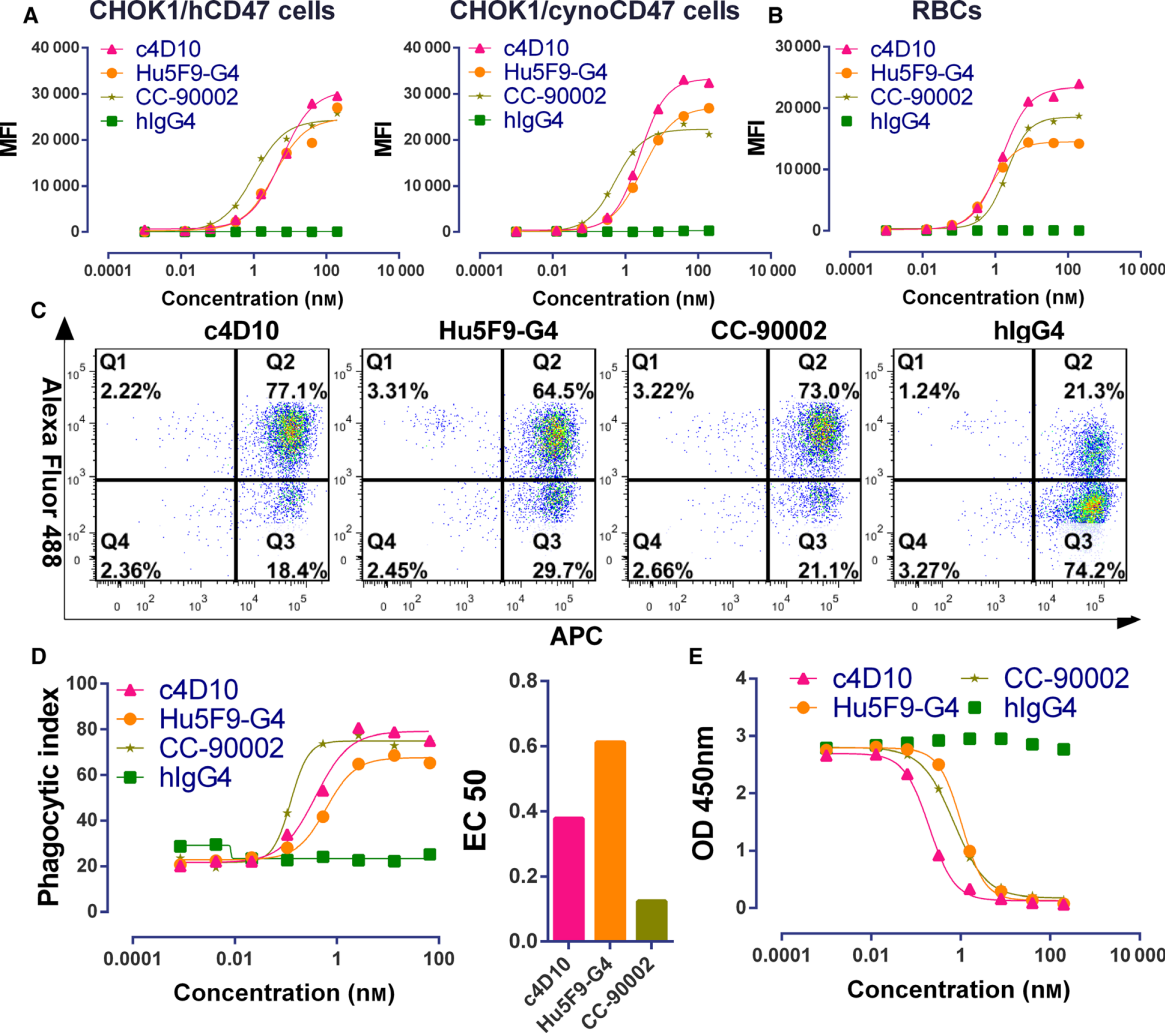
Characterization of c4D10 *in vitro*. (A, B) Antibodies were used to stain CHOK1/cynoCD47 cells (A, left) or CHOK1/hCD47 cells (A, right) or human RBCs (B) prior to detection with Alexa Fluor 488‐conjugated anti‐human secondary antibody by flow cytometry. The experiment was performed three times with similar results being obtained. (C, D) Macrophages were cocultured with CFSE‐labelled Jurkat cells in the presence of hIgG4, c4D10, CC‐90002 or Hu5F9‐G4. Representative cytofluorometric plots reflecting maximum phagocyticity are shown (C), the phagocytosis index was determined by the percentage of Alexa 488^+^ cells within the APC^+^ macrophage cell gate (D, left) and IC_50_ values of indicated antibodies were calculated using prism, version 6 (D, right). Data shown represent *n* = 3 donors. (E) Competitive inhibition of SIRPα binding to CD47. Serial dilutions of c4D10, CC‐90002 and Hu5F9‐G4 disrupt the interaction of CD47 and SIRPα. Results are representative of three independent experiments. All error bars indicate the SEM.

Because there are only three amino acids in the extracellular domain of cynoCD47 that were different from human CD47 and none were involved in the CD47/SIRPα interaction interface, cynomolgus monkeys were utilized widely for preclinical pharmacokinetic and toxicology assessments related to CD47 antibody [28, 29, 30, 31]. Along with CHOK1/hCD47 cells, CHOK1/cynoCD47 cells were employed to detect the binding properties of the three CD47 antibodies. As shown in Fig. 1A, CC‐90002 bound to CHOK1/hCD47 cells and CHOK1/cynoCD47 cells with an EC_50_ of 0.99 nm and 0.54 nm, both being superior to Hu5F9‐G4 (3.73 and 2.96 nm) and c4D10 (5.75 and 2.51 nm). Next, the antigen binding activity of c4D10 to human RBCs was measured to evaluate its possible off‐target effects and preliminary *in vitro* toxicity. The c4D10 dose‐dependently bound human RBCs with an EC_50_ of 1.49 nm, which was slightly stronger than c4D10 (2.0 nm) but two‐fold weaker compare to Hu5F9‐G4 (0.75 nm) (Fig. 1B). To accurately identify the binding activity of c4D10, the Octet Red 384 System, which offers a powerful means of monitoring the molecular interactions of a biomolecular complex in real‐time, was applied to analysed the affinities of three antibodies with hCD47 [32, 33]. The c4D10 antibody bound to recombinant human CD47 antigen with a *K*_D_ of 1.06 nm, which was improved by approximately five‐fold compared to that of CC‐90002 and by 16‐fold compared to that of Hu5F9‐G4 (Table 2).

**Table 2:** Representative data of c4D10 and reference antibodies. NA, not applicable; –, not test. Data are shown as the mean ± SEM.

	c4D10	Hu5F9‐G4	CC‐90002	hIgG4	Vehicle
Affinity					
*K*_D_ (m)	1.06*10^−09^	1.78*10^−08^	5.60*10^−09^	NA	NA
Phagocytosis					
Maximum (index)	79.17	67.65	74.97	29.15	–
EC_50_ (nm)	0.38	0.61	0.12	NA	NA
Haemagglutination					
Max (score)	1.07	2.16	1.19	1.12	–
T‐cell death (66.67 nm)					
Apoptosis (%)	21.5	45.1	30.5	22.9	–
AML xenograft model in NOD/SCID mice					
Tumour volume (mm^3^)	67.44 ± 21.70**	–	10.62 ± 4.32**	–	2543.40 ± 247.03
Tumour weight (g)	0.082 ± 0.025**	–	0.019 ± 0.0068**	–	2.77 ± 0.19
Toxicity study in B‐hSIRPα/hCD47 mice (day 6)					
White blood cells (10^9^·L^–1^)	3.87 ± 0.37	27.09 ± 3.83*	–	22.26 ± 3.00*	4.55 ± 0.32
RBCs (10^9^·L^–1^)	5.53 ± 0.19	3.04 ± 0.33	–	4.11 ± 0.39	6.70 ± 0.44
Mean platelet volume (fl)	4.60 ± 0.08	5.63 ± 0.07**	–	5.47 ± 0.10**	4.37 ± 0.03
CR (μmol·L^−1^)	44.37 ± 8.41	22.36 ± 8.16*	–	23.46.11 ± 6.41	48.92 ± 8.76
UREA (mmol·L^−1^)	1.49 ± 0.13	1.56 ± 0.18*	–	1.45 ± 0.04	1.93 ± 0.21
Animal weight (g)	18.1 ± 0.34	17.47 ± 0.14*	–	19.23 ± 0.38	19.33 ± 0.18

* *P* < 0.05

** *P* < 0.001 versus vehicle. Statistics were performed using one‐way analysis of variance (Tukey–Kramer).

### CD47 lead candidate induces potent macrophage‐mediated phagocytosis of AML

As a critical property of an efficacious antibody, the blocking activity of c4D10 was measured using a competitive ELISA. Surprisingly, the IC_50_ of c4D10 that disrupted the CD47‐SIRPα interaction was 0.20 nm, which was superior to that of Hu5F9‐G4 (1.1 nm) and CC‐90002 (0.71 nm) (Fig. 1E). To further conform the therapeutic potential, we assessed functional phagocytosis mediated by human macrophages in the presence of our candidate and reference antibodies. As shown in Fig. 1D, the three antibodies all strongly promoted the engulfment of Jurkat cells, a T‐cell leukaemia line with high endogenous expression of CD47, in a dose‐dependent manner. The EC_50_ and maximal value of the phagocytic index induced by c4D10 (maximum = 79.17, EC_50_ = 0.37 nm) were to some extent inferior to that of CC‐90002 (maximum = 74.97, EC_50_ = 0.12 nm) but better than that of Hu5F9‐G4 (maximum = 67.65, EC_50_ = 0.61 nm) (Fig. 1C,D). Based on the biochemical behaviour and functional outcome, c4D10 is a potential effective therapeutic antibody worthy of further in‐depth investigation.

### *CD47 lead candidate eradicates primary human AML* in vivo

**Fig. 2:**
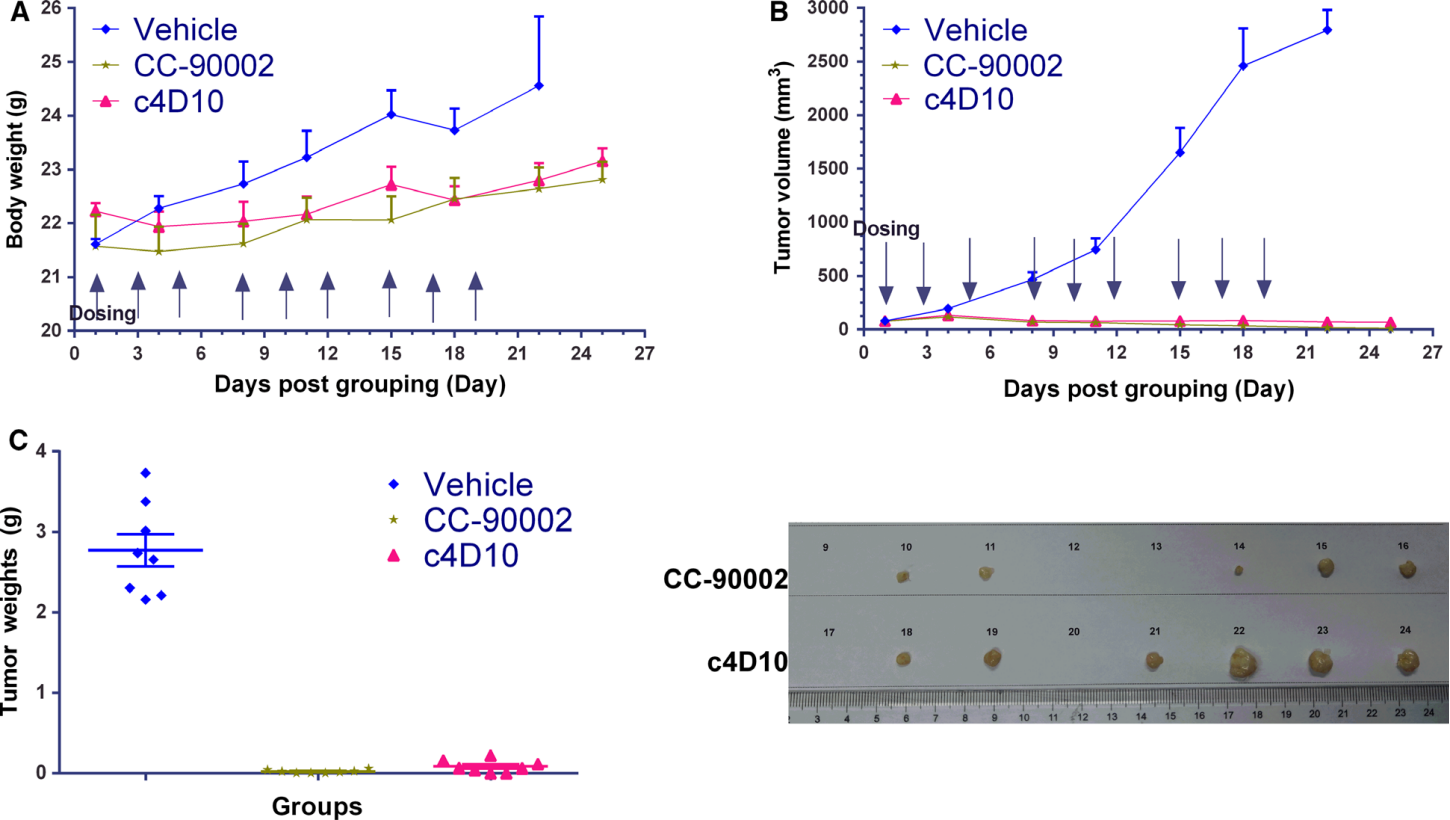
Efficacy study of c4D10 *in vivo*. Raji tumour cells were implanted subcutaneously in the right flank of NOD/SCID mice. Mice with established tumours (average of 79.97 mm^3^) were block randomized and treated with 10 mg·kg^−1^ vehicle, CC‐90002 or c4D10 through an intravenous route on days 1, 3, 5, 8, 10, 12, 15, 17 and 19. The weight of the mice was tested (A) and the tumour size was recorded (B) twice weekly. On day 25 after mice received tumour cells, all mice were killed, and tumours were collected and weighed (C). All error bars indicate the SEM (*n* = 8).

Because SIRPα from NOD/SCID mice bound human CD47 with an exceptionally higher affinity compared to that from other mouse strains, and even greater than hCD47 [34], this strain is an ideal xenograft model for assessing the tumour inhibitory effect of our antibodies. In mice xenografted with the lymphoma cancer cell line Raji, c4D10 and CC‐90002 were simultaneously well tolerated with no obvious body weight lost after correcting by deducting the final tumour weight (Fig. 2A,C). At day 25 post‐inoculation, the mean ± SD tumour volume of CC‐90002‐ and c4D10‐treated mice was 10.62 ± 4.32 and 67.44 ± 21.70 mm^3^, respectively, whereas all mice in the control group were euthanized as a result of large tumour burden (> 2000 mm^3^) within 1 month (Fig. 2B). It is particularly noteworthy that two and three mice achieved almost complete remission throughout treatment with c4D10 and CC‐90002, respectively (Fig. 2C). Although c4D10 was slightly inferior to CC‐90002 with respect to therapeutic efficacy (consistent with previous phagocytic activity data *in vitro*), the two antibodies all displayed robust antitumour immune responses *in vivo*.

### *No adverse event related to c4D10 is observed* in vitro *toxicity studies*

**Fig. 3:**
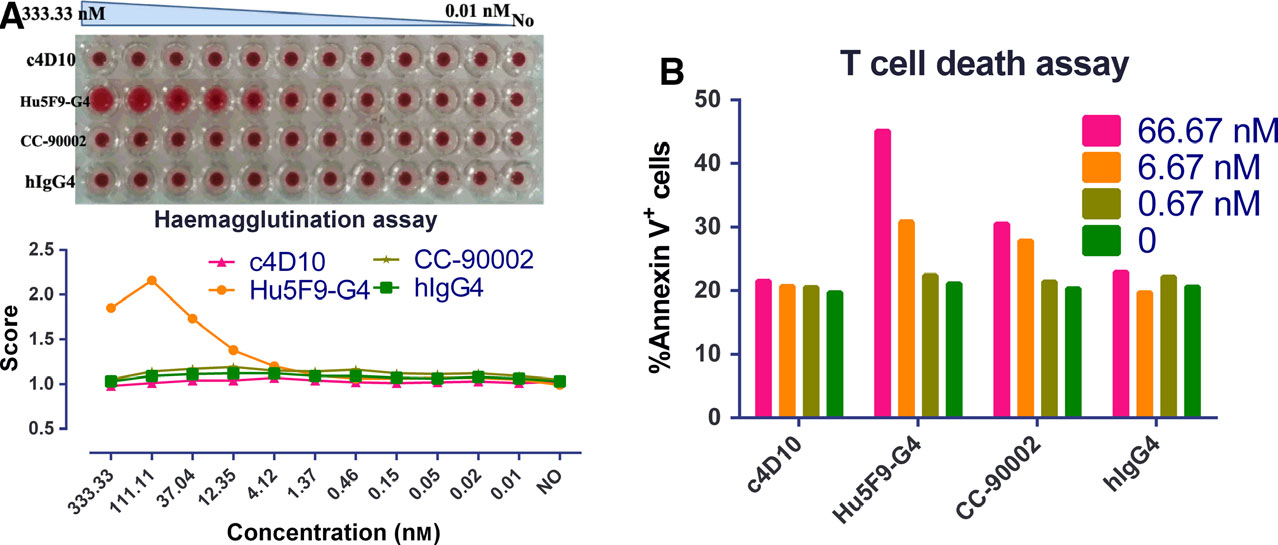
Toxicity study of c4D10 *in vitro*. (A) Haemagglutination assays were conducted with human erythrocytes and titrated amounts of c4D10, CD47‐specific antibodies or hIgG4 (top). The extent of haemagglutination was assessed by the Comparable area of the RBC pellet using Photoshop (Adobe Inc., San Jose, CA, USA) software (bottom). Data shown represent *n* = 3 donors. (B) CD3^+^ cells were cultured with plate‐bound anti‐CD3 in the presence of immobilized anti‐CD47 antibodies overnight prior to detection with Annexin‐V‐Alexa 488 by flow cytometry. Data shown represent *n* = 3 donors.

Because of the high expression level of CD47 on erythrocytes, most CD47 antibodies have been reported to cause haemagglutination of human erythrocytes. A predominant side effect in the use of the antagonist targeting CD47 results from a homotypic interaction, where two CD47‐expressing cells are prone to aggregate or clump together when treated with a bivalent CD47 binding entity [35]. To evaluate the safety of c4D10 as a curative drug, a haemagglutination assay was conducted on human RBCs. In Fig. 3A, c4D10 and CC‐90002 did not induce haemagglutination at any of the concentrations tested; in contrast, Hu5F9 induced haemagglutination of human RBCs with a score of 1.2 or higher at concentrations ranging from 4.12 to 333.33 nm.

Activation‐induced death of T cells plays an important role in the regulation of immune responses. Previous work has demonstrated that engagement of distinct epitopes on CD47 rapidly signals T‐cell death in a novel pathway [36]. In our test (Fig. 3B), severe apoptosis was readily observed in the presence of the reference antibodies, especially Hu5F9‐G4 (maximum = 45.10); in contrast, this phenomenon did not appear in the c4D10 group. From the two toxicity studies conducted *in vitro*, we hypothesized that c4D10 is an anti‐human CD47‐neutralizing antibody providing better safety features.

### Chimeric 4D10 shows good tolerance in hCD47/hSIRPα double knock‐in mice

**Fig. 4:**
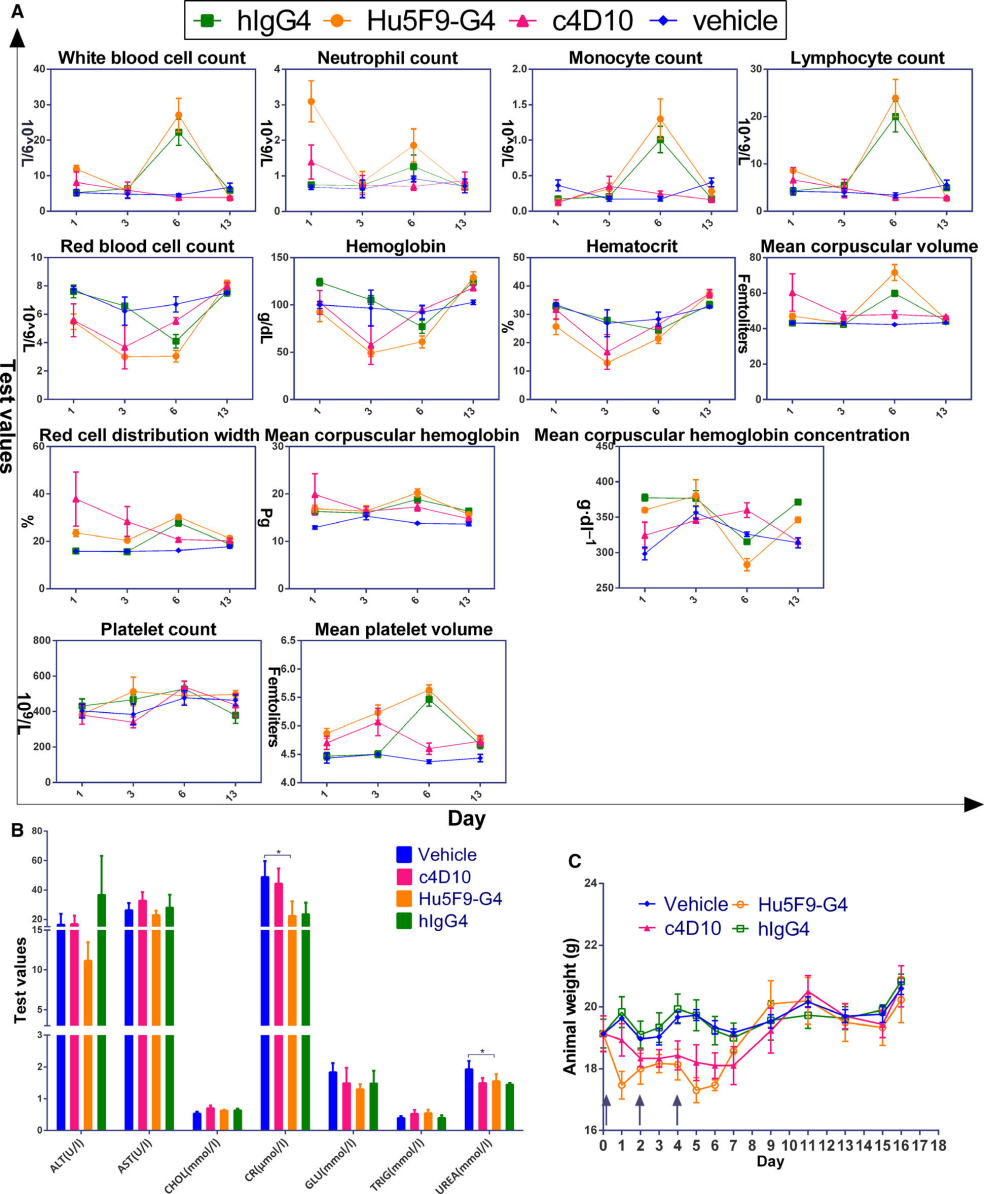
Toxicity study of c4D10 *in vivo*. B‐hSIRPα/hCD47 mice (*n* = 3) were treated with 10 mg·kg^−1^ anti‐CD47 antibodies or control through an intravenous route on days 0, 2 and 4, routine blood tests were performed (A), blood chemistry levels were tested on day 6 (B) and mouse weights were recorded (C). ALT, alanine amino‐transferase; AST, aspartate transaminase; CHOL, cholesterol; CR, creatinine; GLU, glucose; TRIG, triglyceride; UREA, urea. All error bars indicate the SEM (*n* = 3). *P < 0.05. Statistics were performed using one‐way analysis of variance (Tukey–Kramer).

To further detect immune‐related toxicity, a cohort of chimeric B‐hSIRPα/hCD47 mice, comprising an effective *in vivo* model for the development of CD47 and SIRPα antibodies that can be advanced to human clinical trials, was grouped and injected intraperitoneally with the respective antibodies [37]. In the 13‐day observation period, clinical signs, including body weight, complete blood components and serum biochemistry, were monitored. Interestingly, counts of white blood cells (including lymphocytes, monocytes and neutrophils) and mean platelet volume showed a drastic increase at day 6 in the group administered Hu5F9‐G4 or hIgG4, whereas no obvious effect was observed in the group treated with c4D10. At the same time, there was a significant and long‐term reduction of RBC counts, haemoglobin and haematocrit in the Hu5F9‐G4 administered mice with increased mean corpuscular volume and mean corpuscular haemoglobin, suggesting that haemolytic anaemia occurred [38], which was only mild and transient in the c4D10 group (Fig. 4A). In the biochemical analysis, Hu5F9‐G4 also caused a statistically significant decrease in serum creatinine and urea concentrations, which are markers of kidney dysfunction (Fig. 4B) [39]. Because a direct aspect may reflect overall toxicity, animal weight was recorded. Despite there being no difference between the groups with respect to the day by which normal weight was recovered, the group treated with Hu5F9‐G4 clearly displayed a greater weight loss (Fig. 4C).

Briefly, all of these observations indicated that c4D10 was generally well tolerated at a dose of 10 mg·mL^−1^, and no obvious influence on the level of complete blood counts and blood chemistry was observed. Although transient anaemia caused by Hu5F9‐G4 was improved with a priming and maintenance dose regimen [31], long‐term toxicity still needs further follow‐up in more patients and in various malignancies. Overall, c4D10 is a promising antibody that simultaneously possesses desirable therapeutic efficacy and minimal deleterious effects.

## Discussion

As a self‐protection protein, CD47 plays a pivotal role in enabling cancer cells to evade immune elimination [12]. To engage with SIRPα, CD47 transmits a negative signal to macrophages to inhibit phagocytosis and subsequently antigen presentation [10]. Antibodies or other blocking agents, targeting the CD47‐SIRPα axis and blocking the cross‐talk between macrophages and cancer cells, will induce dramatic and durable antitumour immunity by bridging innate and adaptive immune responses [40]. However, as a critical regulator of RBCs, CD47 is functionally involved in the maintenance and clearance of RBCs, suggesting that haemolytic anaemia may occur subsequent to the use of an antagonist targeted towards CD47 [41]. To alleviate this symptom and maximize potency, fusion molecule engineered IgG1 Fc tail and SIRPα proteins were developed, including TTI‐621 and IMM01, which do not bind to RBCs as a result of species‐specific differences in the mobility of CD47 in erythrocyte membranes [42]. Despite the moderate RBC and haemoglobin toxicity compared to the CD47 antibody, the administration of these fusion proteins displayed an overall higher toxicity to other cells resulting from the strong antibody‐dependent cellular cytotoxicity activity of IgG1. In TTI‐621 trials, four of five patients receiving 0.3 mg·kg^−1^ dosages developed G3 and G4 platelet counts, which was not reported in the Hu5F9‐G4 trial [17, 18].

In the present study, we describe the development of c4D10, a chimeric IgG4 subclass antibody that harbours weak binding activity with FcγR and ultrahigh affinity with CD47. In the preclinical pharmacokinetic study, c4D10 effectively inhibited the CD47‐SIRPα interaction, which caused an acute depletion of cancer cells, even with only a limited contribution of antibody‐dependent cellular phagocytosis. Notably, in an *in vivo* toxicity study with hCD47/hSIRPα double knock‐in mice, only transient and reversible anaemia occurred after treatment with c4D10. Furthermore, c4D10 showed relatively high cross‐reactivity with cynomolgus monkey CD47, allowing direct assessment of the safety and toxicokinetic profiles in this non‐human primate, which could potentially be used to inform the design of clinical trials and the starting dose. Taken together, c4D10 is an excellent antagonist with respect to performing effective pharmacokinetics in the elimination of tumour cells at the same time as maintaining a favourable preclinical safety profile (Table 2).

Considering that a pro‐phagocytosis signal is also needed to trigger phagocytosis and that our mAb is an IgG4 subclass antibody [43], it is rational to fuse it with other tumour‐targeting antibodies or other modalities. Previous findings have demonstrated that conventional therapies, such as chemotherapy‐mediated upregulation of cell surface calreticulin and radiotherapy‐induced inflammation, increased the sensitivity of the tumour to macrophages, all of which contributed to the outcomes of anti‐CD47 treatment [44]. Hu5F9‐G4 in combination with the chemotherapeutic azacitidine, the anti‐epidermal growth factor receptor mAb cetuximab and the PD‐L1 targeting antibody avelumab achieved promising outcomes in clinical trials (www.clinicaltrials.gov identifiers: NCT04313881, NCT02953782 and NCT03558139). Although combination strategies with other commercial drugs require further testing, the evidence outlined above firmly suggests that c4D10 is probably an ideal component of a combination strategy and has a similar biological therapeutic potency and lower toxicity compared to Hu5F9‐G4.

Similar to the CD47‐SIRPα axis, malignant cells are capable of avoiding macrophage‐dependent destruction through the overexpression of anti‐phagocytic surface proteins, including programmed cell death ligand 1, β‐2 microglobulin subunit of the major histocompatibility class I complex and CD24 [45, 46, 47]. Interestingly, CD24 acts as a complementary signal of CD47 and appears to have inversely correlated expression in human diffuse large B‐cell lymphoma. Furthermore, ovarian and triple‐negative breast cancers that were particularly susceptible to CD24 blocking did not respond well to CD47‐blocking therapy, and vice versa in leukaemia. In the tumour microenvironment, PD‐1 expression correlates with M2‐polarized macrophages, which play a key role in the growth or regression of tumours. Thus, our research not only focuses on developing possible potential pharmacological applications of c4D10, but also deepens the understanding of homeostatic phagocytosis maintenance with respect to facilitating research on phagocytosis checkpoint blockade. Lastly, the accumulated data in clinical trials provide a significant impetus for the discovery of safe and highly specific phagocytosis checkpoint inhibitors, with c4D10 exhibiting strong clinical value as a drug.

## Conflict of interests

The authors declare the following financial competing interests: ZX, JG, JY and TY are or were full‐time employees of ChemPartner. DW and CD are full‐time employees of Hyamab. Patents pertaining to the results presented in the paper have been filed under the Patent Cooperation Treaty (International Publication Number PCT/CN2019/122777). YD declares no conflict of interests.

## Author contributions

JG, DW, CD and YD designed the experiments. ZX and JY performed the experiments and analysed data. ZX wrote the manuscript. YD reviewed and edited the manuscript. TY supervised the project.

## Data Availability

Data are available from the corresponding author upon reasonable request.
